# Plasma Metabolomics Biosignature According to HIV Stage of Infection, Pace of Disease Progression, Viremia Level and Immunological Response to Treatment

**DOI:** 10.1371/journal.pone.0161920

**Published:** 2016-12-12

**Authors:** Bruno Scarpelini, Michelle Zanoni, Maria Cecilia Araripe Sucupira, Hong-Ha M. Truong, Luiz Mario Ramos Janini, Ismael Dale Cotrin Segurado, Ricardo Sobhie Diaz

**Affiliations:** 1 Federal University of Sao Paulo, Department of Medicine, Sao Paulo—SP, Brazil; 2 University of California at San Francisco, Department of Medicine, San Francisco, CA, United States of America; 3 Federal University of Sao Paulo, Department of Microbiology, Sao Paulo—SP, Brazil; Imperial College London, UNITED KINGDOM

## Abstract

**Background:**

We evaluated plasma samples HIV-infected individuals with different phenotypic profile among five HIV-infected elite controllers and five rapid progressors after recent HIV infection and one year later and from 10 individuals subjected to antiretroviral therapy, five of whom were immunological non-responders (INR), before and after one year of antiretroviral treatment compared to 175 samples from HIV-negative patients. A targeted quantitative tandem mass spectrometry metabolomics approach was used in order to determine plasma metabolomics biosignature that may relate to HIV infection, pace of HIV disease progression, and immunological response to treatment.

**Results:**

Twenty-five unique metabolites were identified, including five metabolites that could distinguish rapid progressors and INRs at baseline. Severe deregulation in acylcarnitine and sphingomyelin metabolism compatible with mitochondrial deficiencies was observed. β-oxidation and sphingosine‐1‐phosphate-phosphatase-1 activity were down-regulated, whereas acyl-alkyl-containing phosphatidylcholines and alkylglyceronephosphate synthase levels were elevated in INRs. Evidence that elite controllers harbor an inborn error of metabolism (late-onset multiple acyl-coenzyme A dehydrogenase deficiency [MADD]) was detected.

**Conclusions:**

Blood-based markers from metabolomics show a very high accuracy of discriminating HIV infection between varieties of controls and have the ability to predict rapid disease progression or poor antiretroviral immunological response. These metabolites can be used as biomarkers of HIV natural evolution or treatment response and provide insight into the mechanisms of the disease.

## Background

The average period for HIV progression from acute infection to AIDS is 8 years[1]. However, elite controllers are able to naturally control HIV-1 replication and maintain adequate CD4+ T cell levels[2], while rapid progressors may evolve to AIDS in a period as short as 2 years[1]. Furthermore, 30% of the HIV-infected population, referred to as immunological non-responders (INR), fail to increase CD4+ T cell counts by at least 30% despite being treated with antiretrovirals and achieving viral suppression for a year or more[3].

Metabolomics, the unbiased identification and quantification of small molecules in biological fluids[4], can serve as a path to the understanding of biochemical state of an organism and aid in the discovery of biomarkers. Furthermore, quantitative measurement by mass spectroscopy of specific metabolic products in plasma, urine or cells from cases compared to those from controls has begun to reveal critical differences in the products of diseased versus normal tissues for a wide variety of conditions, including prostate cancer [5], colon and stomach cancer [6], and Parkinson's disease [7], and HIV. In this regard, profound misbalanced functions related to energy, protein, lipid and glucose metabolism have been reported in HIV-infected individuals since recognition of the disease and introduction of ART [8–12]. Increases in metabolism are reported to be present already during asymptomatic periods and can reach even higher levels during opportunistic infections[10–13]. Very recently, the metabolic pathway related to the transport of the amino acid alanine was proved to be important for T cell activation; Indeed, impairments of alanine transport in CD4 T cells might contribute to HIV-1 pathogenesis through modulation of virus production, weakening of the adaptive immune response as well as enhancement of CD4 T-cell loss [13]. In the current study, we hypothesized that distinct individual phenotype among HIV-infected individuals will display distinct metabolomics profile.

The purpose of this study was to identify metabolites that are unique to HIV-infected individuals and to identify biomarkers that relates to HIV natural evolution and biomarkers that relate to immunological response to antiretroviral treatment using a targeted quantitative tandem mass spectrometry (MS/MS) metabolomics approach in order to gain insights into the mechanisms of HIV.

## Methods

We analyzed four panels of previously unthawed frozen plasma samples from HIV-infected individuals prospectively every 3 months using a targeted quantitative tandem mass spectrometry (MS/MS) metabolomics approach. Twenty patients were selected from a HIV recent infection cohort in Sao Paulo, Brazil. Individuals were identified as recent HIV infections using the Serologic Testing Algorithm for Recent HIV Seroconversion[14]. Written Informed consent has been obtained from all participants and Institutional Review Board (Comitê de Ética em Pesquisa da Universidade Federal de São Paulo / Escola Paulista de Medicina–CEP/UNIFESP-EPM) approved study (#1586/11).

All patients were randomly selected according to their phenotype (elite controllers or rapid progressors) or their response to antiretroviral treatment. Elite controllers were defined as having a viral load below 400 copies/mL plasma after recent infection for a period of at least 2 years, and T+ CD4 cell counts with a positive slope using linear regression (Panel A in S1 Fig). Rapid progressors were defined as having higher viral load positive slopes and a faster decrease in CD4+ T cell counts using linear regression (Panel B in S1 Fig). Selected patients were not using any concurrent medications or supplements, did not have any detected comorbidities, and did not have any laboratory abnormalities related to blood cell counts, glycose, liver, kidney or pancreatic measurements.

Group A comprised samples from five elite controllers collected during recent HIV infection (Panel A1 in S1 Fig) and after one year of follow-up (Panel A2 in S1 Fig). Group B (in S1 Fig) used the same strategy for 5 HIV-1 rapid progressors, with samples collected during recent HIV infection (Panel B1 in S1 Fig) and after one year of follow-up (Panel B2 in S1 Fig). Group C consisted of five patients who underwent antiretroviral therapy after reaching CD4+ T cell counts below 350 cells/ μ L, and in whom viral loads reached levels below detection limits of 50 copies/mL and CD4+ T cell counts increased to at least 30% from baseline upon treatment. Group D used the same strategy for five INR. Antiretroviral treatment on groups C and D was homogeneously comprised of an association of fixed dose combination of zidovudine and 3TC administered BID, and a QD dose of Efavirenz, according to the local Brazilian guidelines at that time. We analyzed samples from patients who experienced different paces of disease progression (Group A versus Group B) compared to patients who were either viremic (Groups B, C1 and D1), naturally aviremic (Group A), aviremic upon antiretroviral treatment (Group C2 and D2), or presented a distinct immunological response upon treatment (groups C versus D). The clinical characteristics of the subjects are depicted in Table A in S1 File.

All metabolomics data were used as received from Biocrates. Samples were blindly analyzed, and no data points were removed. The experimental metabolomics measurement technique is described in detail by US patent 2007/0004044 (accessible online at Free Patents Online). The company had no access to phenotype information that would have permitted any data pre-filtering other than objective quality control for measurement errors based on internal controls and duplicates.

A summary of the method can be found in [15–17] and a comprehensive overview of the field and the related technologies is given in the review paper by Wenk[18]. Briefly, a targeted profiling scheme was used to quantitatively screen for known small molecule metabolites using multiple reaction monitoring, neutral loss and precursor ion scans. Quantification of the metabolites of the biological sample was achieved by referencing to appropriate internal standards. The method is in conformance with 21CFR (Code of Federal Regulations) Part 11, which implies proof of reproducibility within a given error range. The concentrations of all analyzed metabolites were reported in μM and the results were compared to tumor response rates and tumor intrinsic subtypes. This method has been used in different academic and industrial applications[19].

The metabolite panel is composed of 186 different metabolites: 40 acylcarnitines, 19 proteinogenic amino acids, ornithine and citrulline, 19 biogenic amines, the sum of hexoses, 76 phosphatidylcholines, 14 lyso-phosphatidylcholines and 15 sphingomyelins.

Glycerophospholipids are further differentiated with respect to the presence of ester (a) and ether (e) bonds in the glycerol moiety, where two letters (aa = diacyl, ae = acyl-alkyl, ee = dialkyl) denote that two glycerol positions are bound to a fatty acid residue, while a single letter (a = acyl or e = alkyl) indicates the presence of a single fatty acid residue.

Lipid side chain composition is abbreviated as Cx:y, where x denotes the number of carbons in the side chain and y the number of double bonds. For example, ‘‘PC ae C38:1” denotes a plasmalogen/plasminogen phosphatidylcholine with 38 carbons in the two fatty acid side chains and a single double bond in one of them.

In addition to individual quantification, groups of metabolites related to specific functions were analyzed. Groups of AAs were computed by summing the levels of AA belonging to certain families or chemical structures depending on their functions such as essential AA, non-essential AA, glucogenic AA, total AA, branched-chain AA, Aromatic AA, glutaminolysis AA (Ala+Asp+Glu). Groups of ACs, important to evaluate mitochondrial function, were also computed by summing (Total AC, C2+C3, C16+C18, C16+C18:1, C16-OH+C18:1-OH). Groups of lipids, important to evaluate lipid metabolism, were also analyzed by summing (total LPCs, total PC aa, total PC ae, total SMs, total lipids) (Table B in S1 File).

Proportions among metabolites such as the Fischer’s ratio, a clinical indicator of liver metabolism and function [13] or the clinical indicators of isovaleric acidemia, tyrosinemia and urea cycle deficiency were calculated, as the ratios of branched chain amino acid (leucine+isoleucine+valine) to aromatic amino acid (tyrosine+phenylalanine), valerylcarnitine to butyrylcarnitine (C5/C4), tyrosine to serine (Tyr/Ser) respectively. A complete list of ratios reflecting enzyme activities of specific metabolic pathways have been previously described. [5]

To unambiguously identify and quantify metabolites, stable isotope dilution-multiple reaction monitoring mass spectrometry was performed using targeted quantitative metabolomics platforms at Biocrates (Life Sciences AG, Innsbruck, Austria) in 215 plasma samples; 40 from HIV patients and 175 from controls (58 healthy volunteers, 53 colon cancer patients and 64 breast cancer patients, because the metabolic profile of activated inflammatory cells is similar to tumor cells[20]).Multivariate profile-wide predictive models were constructed using Cross Validated Partial Least Squares Discriminant Analysis (PLS-DA). For each metabolite, the data were mean centered and scaled to unit variance[21]. Associations between the 28 blood metabolites and HIV-1 infection were assessed using Pearson’s r analysis. We do not report in this study the metabolite profiles that are common between the study group and control groups, but only metabolites that were unique to HIV infection.

The number of latent variables in each model was selected using stratified 10-fold cross validation and calculating associated R2 and Q2 statistics. The predictors were subjected to permutation testing. The results (p<5e-04) confirmed our PLS-DA analysis and revealed a clear discrimination between plasma samples from 40 samples from 20 HIV-infected individuals and 175 HIV negative counterparts employing PLS-DA and permutation testing analysis (p<5e-04 after 2000 permutations) (S2 Fig). Receiver operating characteristic (ROC) curves were determined during training and validation sets such that an accurate assessment of discriminatory ability could be made confirming the existence of highly discriminative metabolites.

Training cases were used for marker discovery and to identify any clinical variable that might be associated with a response by logistic regression analysis. Quantification of metabolite concentrations and quality control assessment was performed with the MetIQ software package (BIOCRATES Life Sciences AG, Innsbruck, Austria). Internal standards served as the reference for the metabolite concentration calculations. An Excel file was then exported, which contained sample names, metabolite names and metabolite concentration with the unit of μmol/L of plasma.

For metabolomic data analysis, log-transformation was applied to all quantified metabolites to normalize the concentration distributions. The data were uploaded into the web-based analytical pipeline MetaboAnalyst 2.0 (MetaboAnalyst) and normalized using MetaboAnalyst normalization protocols[22] for uni- and multivariate analysis, high dimensional feature selection, clustering and supervised classification, functional enrichment and metabolic pathway analysis. Significantly altered metabolites were defined by a T Test analysis with p-value <0.05 and FDR ≤0.05.

The data were also imported to ROCCET (ROC Curve Explorer & Tester; available at ROCCET) for the generation of uni- and multivariate Receiver Operating Characteristic (ROC) curves obtained through Support Vector Machine (SVM), Partial Least Squares-Discriminant Analysis (PLS-DA) and Random Forests.

Curves were generated by Monte-Carlo cross validation (MCCV) using balanced subsampling where two thirds (2/3) of the samples were used to evaluate the feature importance. Significant features were then used to build classification models that were validated on the remaining 1/3 of the samples. The same procedure was repeated multiple times to calculate the performance and confidence interval of each model.

## Results and Discussion

**Fig 1:**
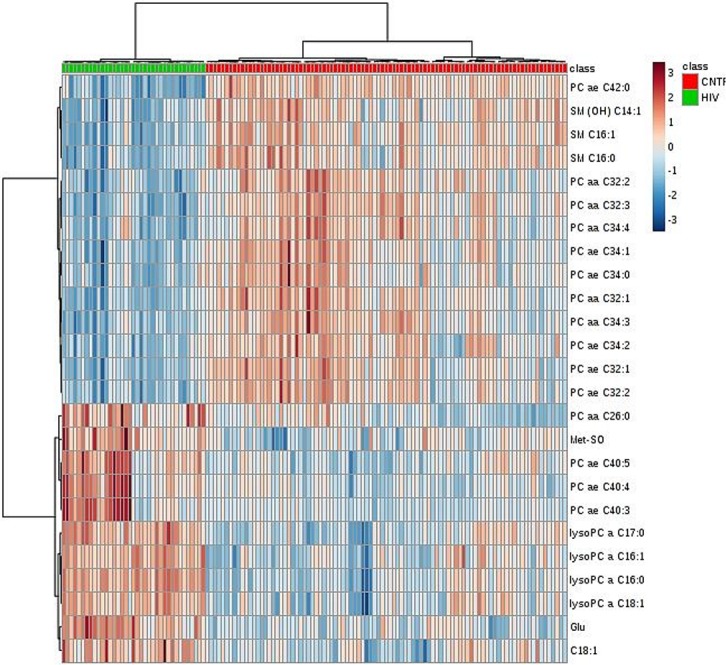
Unsupervised Heat map Clustering Analysis depicting identification of metabolites able to differentiate between Cases and Controls.

A descriptive analysis of 28 blood metabolites and their correlation with HIV-1 infection is shown in Table 1 (and S3 Fig). Unsupervised multivariate analysis using Heat Map (Fig 1) and Randon Forest classification were also conducted between cases and controls. Results demonstrated the existence of metabolites whose blood concentrations can clearly differentiate controls from patients either on acute or chronic phases. The out of the box (OOB) error, after 5000 trees, is 0.0 according Random Forest classification.

**Table 1:** The top 28 metabolites whose concentrations were statistically elevated or decreased in HIV patients compared to controls. FDR = False Discovery Rate; C5-M-DC = Methylglutarylcarnitine; lysoPC a C24:0 = Glycerophospholipids; C5:1-DC = Glutaconylcarnitine; PC aa C42:5 = Glycerophospholipids; lysoPC a C14:0 = Glycerophospholipids; PC aa C30:0 = Glycerophospholipids; Phosphatidylcholines PC aa C28:1 = Glycerophospholipids; C12-DC = Dodecanedioylcarnitine; SM = Sphingomyelin.

Metabolite	Correlation	T test	pValue	FDR
PC ae C38:1	0.84706	10.205	8.0647E-13	1.9306E-5
C5-M-DC	0.84089	99.488	1.71E-08	2.01E-07
lysoPC a C24:0	0.82279	92.698	1.29E-07	1.44E-06
C5:1-DC	0.79126	82.856	2.69E-06	2.68E-05
Glutamate	0.71738	65.935	6.20E-04	5.58E-03
PC ae C40:3	0.57136	4.4578	6.2893E-5	1.082E-11
Aspartate	0.7492	72.427	7.50E-05	7.09E-04
PC aa C42:5	0.6687	57.588	9.53E-03	8.19E-02
SM C26:0	-0.57777	-4.5326	4.9721E-05	3.0639E-4
lysoPC a C14:0	-0.63814	-53.072	4.15E-02	3.14E-02
PC aa C30:0	-0.64955	-54.703	2.44E-02	1.92E-01
PC aa C28:1	-0.66539	-57.075	1.13E-03	9.26E-02
SM C26:1	-0.79524	-83.987	1.89E-06	1.99E-06
C12-DC	-0.8409	-99.493	1.70E-08	2.01E-07
SM C20:2	-0.8444	-10.093	1.12E-08	1.51E-11
Nitrotyrosine	-0.86159	-10.869	1.20E-10	1.75E-08
Dopamine	-0.86968	-11.282	3.79E-11	5.97E-10
SM C18:1	-0.87877	-11.791	9.42E-11	1.62E-09
SM C18:0	-0.88526	-12.187	3.26E-12	6.15E-10
SM (OH) C16:1	-0.89156	-12.605	1.09E-11	2.28E-10
SM C16:0	-0.90533	-13.649	7.69E-13	1.82E-11
SM (OH) C24:1	-0.91078	-14.124	2.40E-13	6.49E-12
SM (OH) C14:1	-0.91275	-14.307	1.55E-13	4.88E-12
SM C24:1	-0.91696	-14.716	5.85E-14	2.21E-12
SM C16:1	-0.92619	-15.729	5.71E-15	2.70E-13
SM (OH) C22:1	-0.93876	-17.445	1.40E-16	8.83E-15
SM (OH) C22:2	-0.94566	-18.622	1.29E-17	1.22E-15
SM C24:0	-0.94912	-19.298	3.47E-19	6.56E-16

Very low concentrations of sphingomyelins and dopamine in parallel with high levels of dicarboxylicacylcarnitines, L-aspartate and many plasmalogen/plasminogen phosphatidylcholines, such as PC ae C38:1 and PC ae C40:3, were detected in the blood of HIV-1-infected individuals compared with controls.

The severe deregulation in acylcarnitine and sphingomyelin metabolism suggests that HIV infection leads to deficiencies in mitochondrial function. Therefore, we assembled ratios of certain metabolite concentrations as proxies for enzymatic activity. We examined the proportion of esterified to free carnitines, β- and Ω-oxidation, and the rate-limiting step in the uptake of fatty acids into the mitochondria related to carnitine palmitoyl transferase I (CPT1) activity. We also examined the SYNE2 locus because of its relation to *SGPP1* (sphingosine‐1‐phosphate phosphatase 1) activity, a key player in the sphingosine rheostat that governs the interchange between pro-apoptotic ceramides and S1P, a well-established ligand in survival signaling[23].

**Fig 2:**
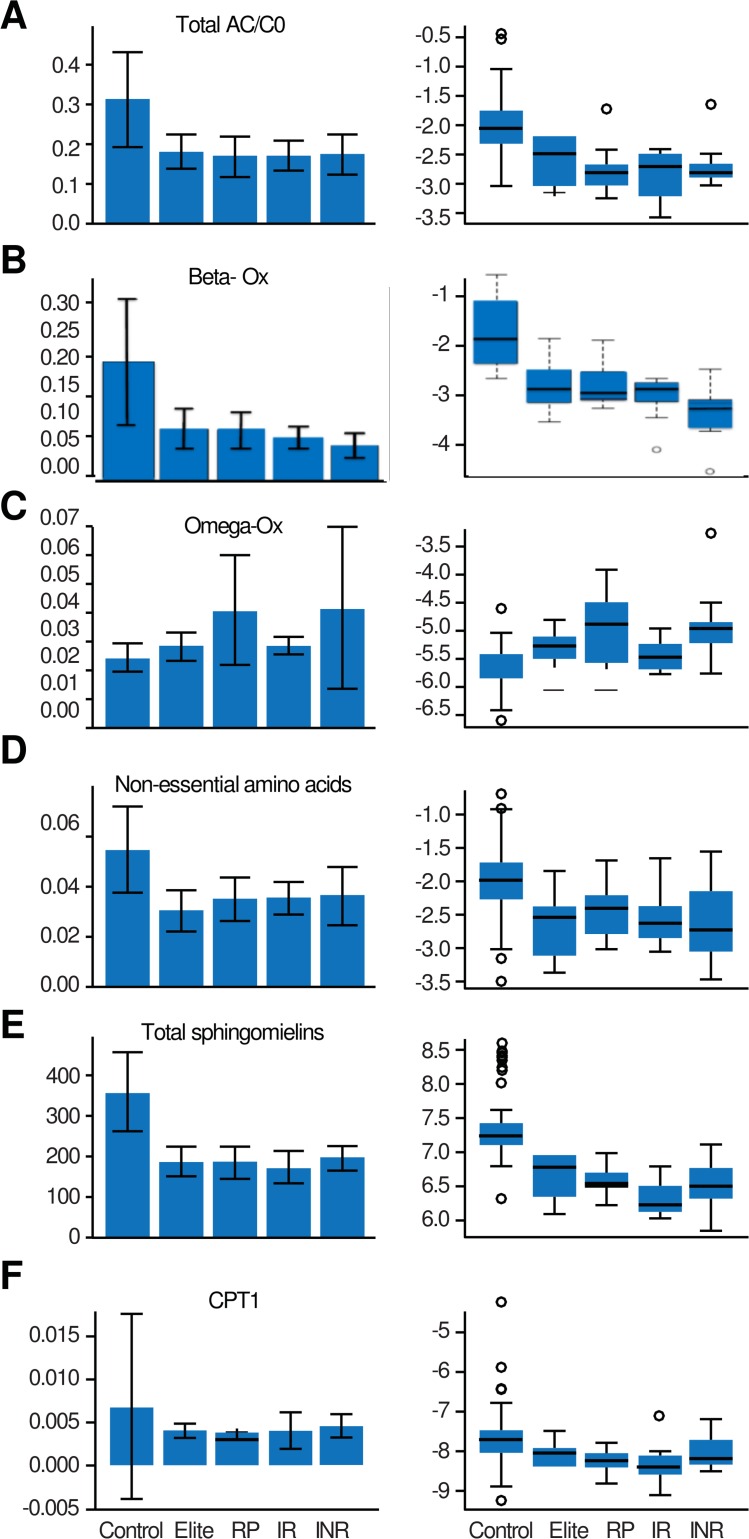
Proportion of Esterified to free Carnitines (Total AC/C0) (A; p = 9.8245E-11 and FDR = 4.1977–10), Beta oxidation (B; p = 1.3529E-13 and FDR = 8.4782E-13) Omega Oxidation (C; p = 6.9445E-11 and FDR = 3.1085E-10), Non-Essential Amino acids other than Glu and Asp (D; p = 1.5306E-47 and FDR = 7.1938E-46), sphingomyelin (E; p = 1.1088E-18 and FDR = 6.74E-19) and uptake of fatty acids to mitochondria (CPT1) (F; p = 0.0016126 and FDR = 0.0026136). Y Axis is depicting micro molar plasma concentrations. Cont = controls; Elite = elite controllers; RP = rapid progressors; IR = immunological responders; INR = immunological non-responders.

ANOVA statistical analysis confirmed our hypothesis by demonstrating that HIV infection is associated with a substantial deterioration in mitochondrial function. This conclusion is supported by a decrease in the proportion between esterified and free carnitines ((Total esterified carnitines(AC)/ free carnitines (C0)) (p = 9.8245E-11 and False Discovery Rate (FDR) = 4.1977–10) (Fig 2A), decreased β-oxidation (p = 1.3529E-13 and FDR = 8.4782E-13) (Fig 2B) in parallel with increased Ω-oxidation (p = 6.9445E-11 and FDR = 3.1085E-10) (Fig 2C), and decreased uptake of fatty acids by the mitochondria (CPT1) (p = 0.0016126 and FDR = 0.0026136) (Fig 2F). As a consequence, the direct products of normal mitochondria, such as non-essential amino acids (p = 1.5306E-47 and FDR = 7.1938E-46) (Fig 2D) and sphingomyelins (p = 1.1088E-18 and FDR = 6.74E-19) (Fig 2E) were down-regulated in patients with HIV (Fig 2A to 2F). Disturbances in fatty acid oxidation (FAO), as revealed by declines in CPT1 and β-oxidation functions, were recently reported to be very important in T cell survival and the promotion of CD8+ TM cell development[24]. Furthermore, it has been shown that perturbations on sphingolipids and glycerophospholipids altering membrane lipid composition may impair innate immune responses.[25] As depicted in Fig 2B, β-oxidation is particularly down-regulated (p = 2.5195E-8; FDR = 1.1412E-7) among INR.

**Fig 3:**
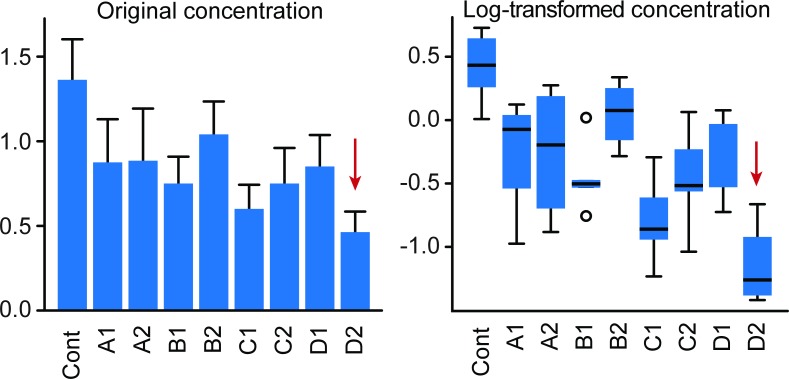
Histogram representing the mean Sphingosine‐1‐Phosphate Phosphatase 1 activity (ratio SGPP1) in different groups. Sphingolipid metabolism is decreased in HIV patients compared to healthy controls (T Test = 0.012128) particularly in the INR group after antiretroviral treatment (arrow) as demonstrated by the ANOVA analysis of SGPP1 activity (p = 2.5266E-7, -log10(p) = 6.5975, FDR = 2.8123E-6). Y Axis is depicting micro molar plasma concentrations. Cont = controls; A1 and A2 are Elite controlers during recent infection and after one year of enrollment; B1 and B2 = Rapid Progressors during recent infection and after one year of enrollment; C1 and C2 = Immunologic Responders before treatment and after one year of treatment, D1 and D2 = INR before treatment and after one year of treatment.

Furthermore, there was a significant decline in sphingosine‐1‐phosphate phosphatase 1 activity (SGPP1, SYNE2 locus) after treatment, particularly among INR, when evaluated by the ratio PC aa C28:1/PC ae C40:2 (p = 8.4667E-7, -log10(p) = 6.0723, FDR = 1.2712E-5) (Fig 3). Importantly, Sphingosine-1-Phosphate (S1P) is involved in lymphocyte egress from lymphoid organs[26, 27] and bone marrow[28, 29] into circulatory fluids via a gradient of S1P. Because SGPP1 (SYNE2 Locus) is correlated to CD4+ T cell counts (p = 0.0071195; FDR = 0.16446, Fig 3), it is tempting to speculate the existence of a link between Sphingosine‐1‐Phosphate Phosphatase 1 activity and INR.

**Fig 4:**
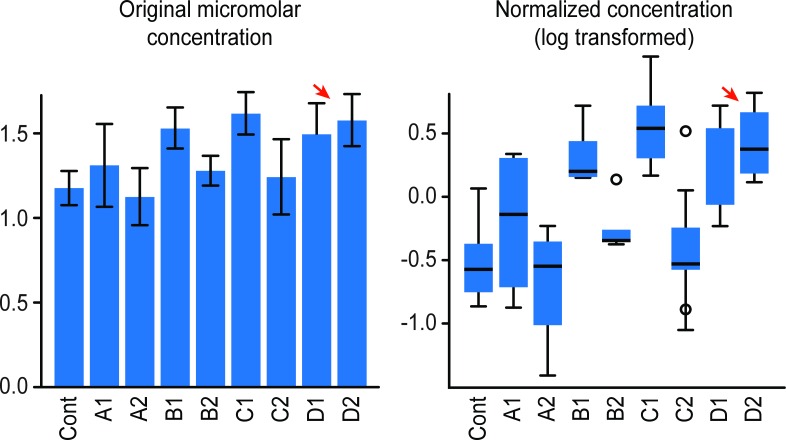
Histogram representing the mean Ether lipid concentration in different groups. Ether lipid production returns to normal levels after 1 year of follow-up except in the INR group (arrow, p = 1.1405E-5, -log10(p) = 4.9429, FDR = 9.6586E-5). Y Axis is depicting micro molar plasma concentrations. Cont = controls; A1 and A2 are Elite controllers during recent infection and after one year of enrollment; B1 and B2 = Rapid Progressors during recent infection and after one year of enrollment; C1 and C2 = Immunologic Responders before treatment and after one year of treatment; D1 and D2 = INR before treatment and after one year of treatment.

The amount of ether lipids as measured by the total acyl-alkyl-containing phosphatidylcholines to total phosphatidylcholines (AGPS) ratio was down-regulated after 1 year of follow-up in all groups but INR (p = 1.1405E-5, -log10(p) = 4.9429, FDR = 9.6586E-5, Fig 4). Because ether lipids activate thymic and peripheral semi-invariant natural killer T cells known to be evolutionarily conserved lipid reactive T cells, we hypothesized that the metabolic enzyme alkylglycerone phosphate synthase (AGPS), a critical step in the synthesis of ether lipids, could be aberrantly activated among INR, leading to impaired CD4+ T cell recovery. We therefore evaluated ether lipid biosynthesis activity after treatment *vis a vis* viral load level and CD4/CD8 in all patients who naturally control viremia (Elite controllers) or Immunological Responders. The results revealed a significant negative correlation (p = 8.5025E-7; FDR = 1.1053E-4) between Ether Lipids (AGPS) and increasing levels of CD4 (from 160 to 1215 mm3) (PostHoc = 160 > 1215; 361 > 1215), with opposite results observed for increases in viral load (p = 8.5025E-7 –Log10(p) = 4.9429, FDR = 1.1053E-4). In addition, the amount of ether lipids remains elevated among INR even during periods of undetectable viral load (p = 1.1537E-4, FDR = 3.5435E-4) when significant declines in SGPP1 (p = 1.0626E-20, FDR = 3.046E-19) and in β-Oxidation (p = 3.3247E-5,FDR = 1.0212E-4) are also observed. Lipid alterations in HIV-infected individuals receiving protease inhibitors based antiretroviral treatment determined using untargeted metabolomic profiling of plasma, has been previously linked to markers of inflammation, microbial translocation, and hepatic function, suggesting that dysregulated innate immune activation and hepatic dysfunction are occurring among HIV antiretrovirally-treated individuals[11]. Furthermore, metabolomic profile in HIV-infected children shows hypoleptinemia and hypoadiponectinemia and is the activation of critical adipose tissue storage and function in the adaptation to malnutrition[30]. Also, alterations in the Cerebrospinal fluid metabolome among HIV antiretrovirally-treated individuals harboring HIV-associated neurocognitive disorders reveal that persistent inflammation, glial responses, glutamate neurotoxicity, and altered brain waste disposal are associated with cognitive alteration[31]. In the current study, we did not assess markers for microbial translocation or the nutritional status of recruited patients. We were not able to find any difference in the metabolomics profile between gender and age, and this may be attributed to the small sample size of this study, since only 3 women were included and only three individuals were over 50 years old out of 20 participants.

We investigated the presence of a metabolomic signature that can be used to identify “Rapid Progression” and “INR” at baseline. As seen in S1 Fig, a combination of five different metabolites and ratios were able to accurately identify Rapid Progressors or INR at baseline with 88.89% sensitivity, 92.31% specificity, 88.89% positive predictive value and 92.31% negative predictive value (AUC = 0.871; 95% CI: 0.619–1; p = 0.01). During the discovery phase, the results repeatedly pointed to metabolites and ratios linked to metabolism affecting acylcarnitine hydroxylation and carboxylation as well as the catabolism of branched chain amino acids, lysine, organic acids, and tryptophan (Table 1). Notably, when elevated, as seen among Elite controllers, these biochemical markers are highly suggestive of an inborn error of metabolism named late-onset multiple acyl-coenzyme A dehydrogenase deficiency (MADD, MIM#231680)[32]. Therefore, we quantified the amount of organic acids, branched chain amino acids and lysine as a diagnostic approach for MADD[32], in addition to using the ratio C7-DC/C8 as a proxy to analyze the activity of a MADD related enzyme, electron-transferring flavoprotein dehydrogenase (ETFDH). The results demonstrated increased levels of alpha aminoadipic acid (p = 0.029658, -log10(p) = 1.5279, FDR = 0.078855), lysine (p = 0.02768, -log10(p) = 1.5578, FDR = 0.075369) and Branch Chain Amino Acids (BCAA) (p = 3.2721E-12, -log10(p) = 11.485, FDR = 1.6189E-11) among Elite controllers. Moreover, the ETFDH activity is significantly less active among Elite controllers compared to the other HIV-infected groups (T-Test = 6.505E-4) and to HIV-uninfected controls (T-Test = 0.0092744). Therefore, possibly an inborn error of metabolism (MADD) and its reduction of ETFDH activity, which can be asymptomatic in many individuals, relates to a control of HIV replication and a functional cure of HIV infection. In order to tease out if any metabolomics profile were related to antiretroviral use, we compared the profile of samples from individuals under antiretroviral therapy (last time point of immunological responders and IRN) with samples from other patients/time points, and we have not been able to identify any specific signature that might be related to antiretroviral use (data on file).

Suppressing HIV replication using antiretrovirals does not completely abrogate the accelerated tissue and organ damage among HIV-infected individuals compared to HIV-uninfected controls[33]. The results presented here make it clear that in addition to their utility as reliable biomarkers, metabolomic profiles of HIV-infected individuals can provide insights into mechanisms of HIV-related tissue and organ damage, and further the development of interventional strategies, such as fixing the decrease levels of dopamine seen among HIV-infected individuals in this study. Of note, low dopamine levels have been implicated in the mechanisms of psychiatric diseases such as depression [34–36] and schizophrenia[37]. As an example and corroborating the predicative abilities of the metabolic signatures identified in blood collected at baseline, of patients that years later developed specific HIV phenotypes, a recent study have been able to identify functional annotations that accurately predicted the inflammatory response of cells derived from patients suffering from inborn errors of metabolism solely on their altered membrane lipid composition[25].

We recognize that further external validation with larger groups of patients is necessary to consolidate the results presented here. Furthermore, metabolites among HIV-infected individuals should also be adjusted by clinical manifestation, biochemistry parameters, cytokines profiles, cell activation, apoptosis and translocation markers, etc.

## Conclusions

To the best of our knowledge, this is the first description of blood-based markers from metabolomics showing a very high accuracy of discriminating HIV infection between a variety of controls and also have the ability to predict rapid disease progression or poor antiretroviral immunological response. These data suggest that the metabolites evaluated here can be used as biomarkers of HIV natural evolution or treatment response and provide insight into the mechanisms of the disease.

## Supporting Information

S1 Fig Viral load and CD4+ T cell count linear regression from a period before 2 years of follow up in elite controllers (Panel A) and rapid progressors (panel B).
(TIF)

S2 Fig PLS-DA and permutation testing analysis (p<5e-04 after 2000 permutations) revealing clear differences between patients with HIV and controls. (TIF)

S3 Fig Comparison between the multivariate ROC curves obtained in training and validation sets using the 5 HIV predictive metabolites described in Table 1. The empirical p values after 100 permutation rounds are also shown.
(TIF)

S1 File Supplementary tables A and B.
(DOCX)
