# Correction: Plasma Metabolomics Biosignature According to HIV Stage of Infection, Pace of Disease Progression, Viremia Level and Immunological Response to Treatment

**DOI:** 10.1371/journal.pone.0173164

**Published:** 2017-02-24

**Authors:** Bruno Scarpellini, Michelle Zanoni, Maria Cecilia Araripe Sucupira, Hong-Ha M. Truong, Luiz Mario Ramos Janini, Ismael Dale da Silva, Ricardo Sobhie Diaz

The first author’s name is spelled incorrectly. The correct name is: Bruno Scarpellini. The sixth author’s name is also spelled incorrectly. The correct name is: Ismael Dale da Silva. The correct citation is: Scarpellini B, Zanoni M, Sucupira MCA, Truong H-HM, Janini LMR, da Silva ID, et al. (2016) Plasma Metabolomics Biosignature According to HIV Stage of Infection, Pace of Disease Progression, Viremia Level and Immunological Response to Treatment. PLoS ONE 11(12): e0161920. doi:10.1371/journal.pone.0161920
