# Supplementary figures and images for: Plasma Metabolomics Biosignature According to HIV Stage of Infection, Pace of Disease Progression, Viremia Level and Immunological Response to Treatment
Source: PLoS One. 2016 Dec 12;11(12):e0161920. doi: 10.1371/journal.pone.0161920 (PMC5152829; doi:10.1371/journal.pone.0161920)

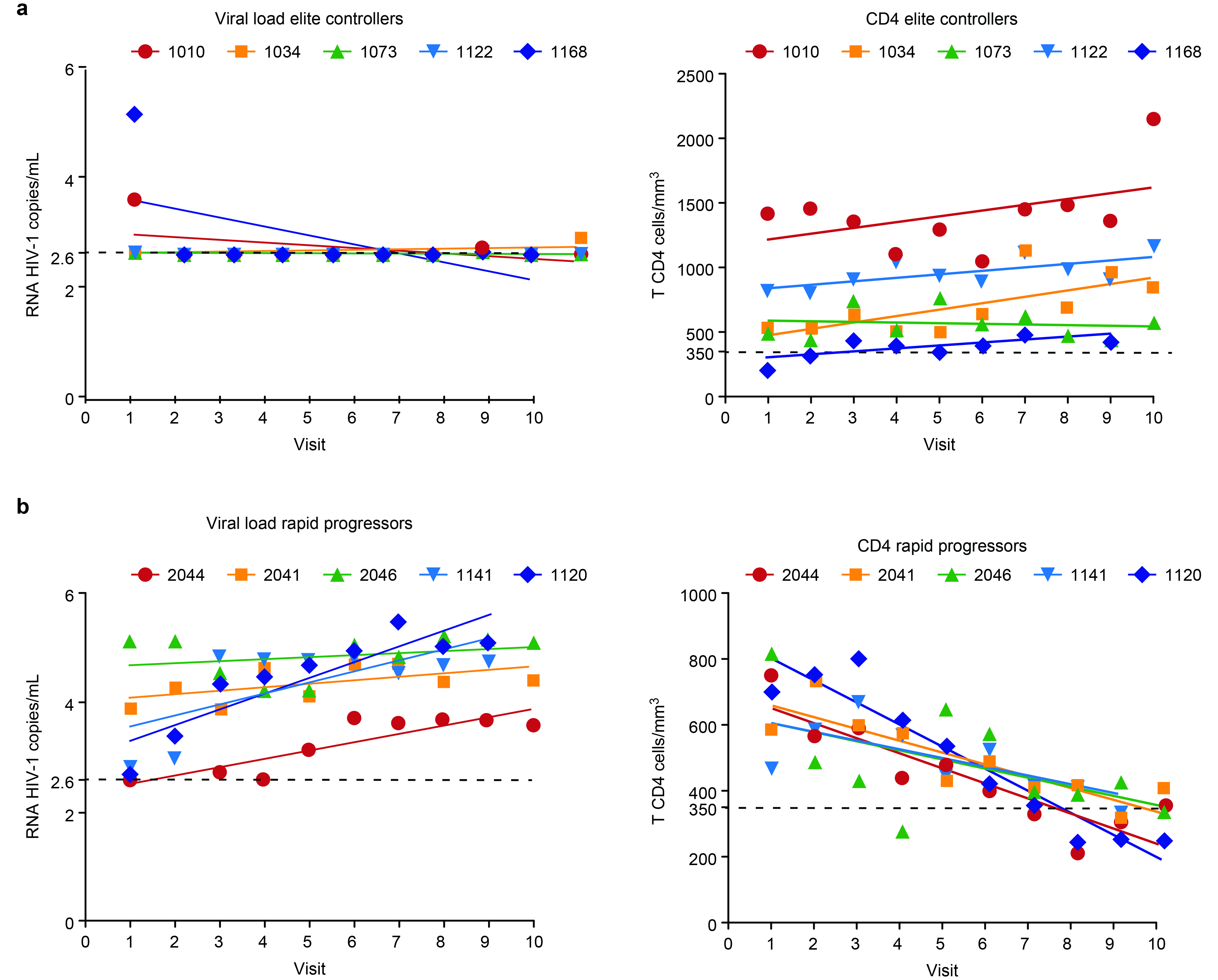

Supplement: S1 Fig — Viral load and CD4+ T cell count linear regression from a period before 2 years of follow up in elite controllers (Panel A) and rapid progressors (panel B). (TIF) [file pone.0161920.s001.tif]

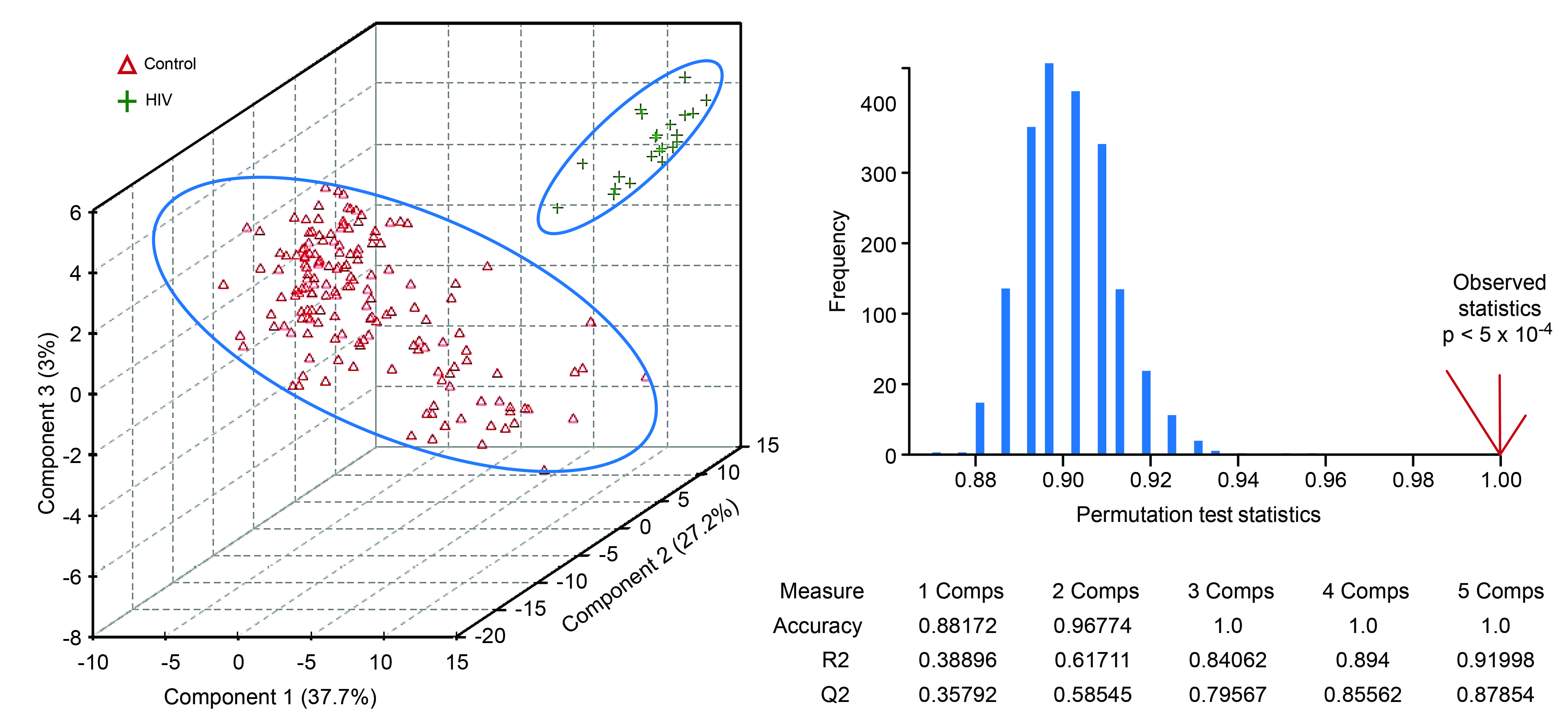

Supplement: S2 Fig — (TIF) [file pone.0161920.s002.tif]

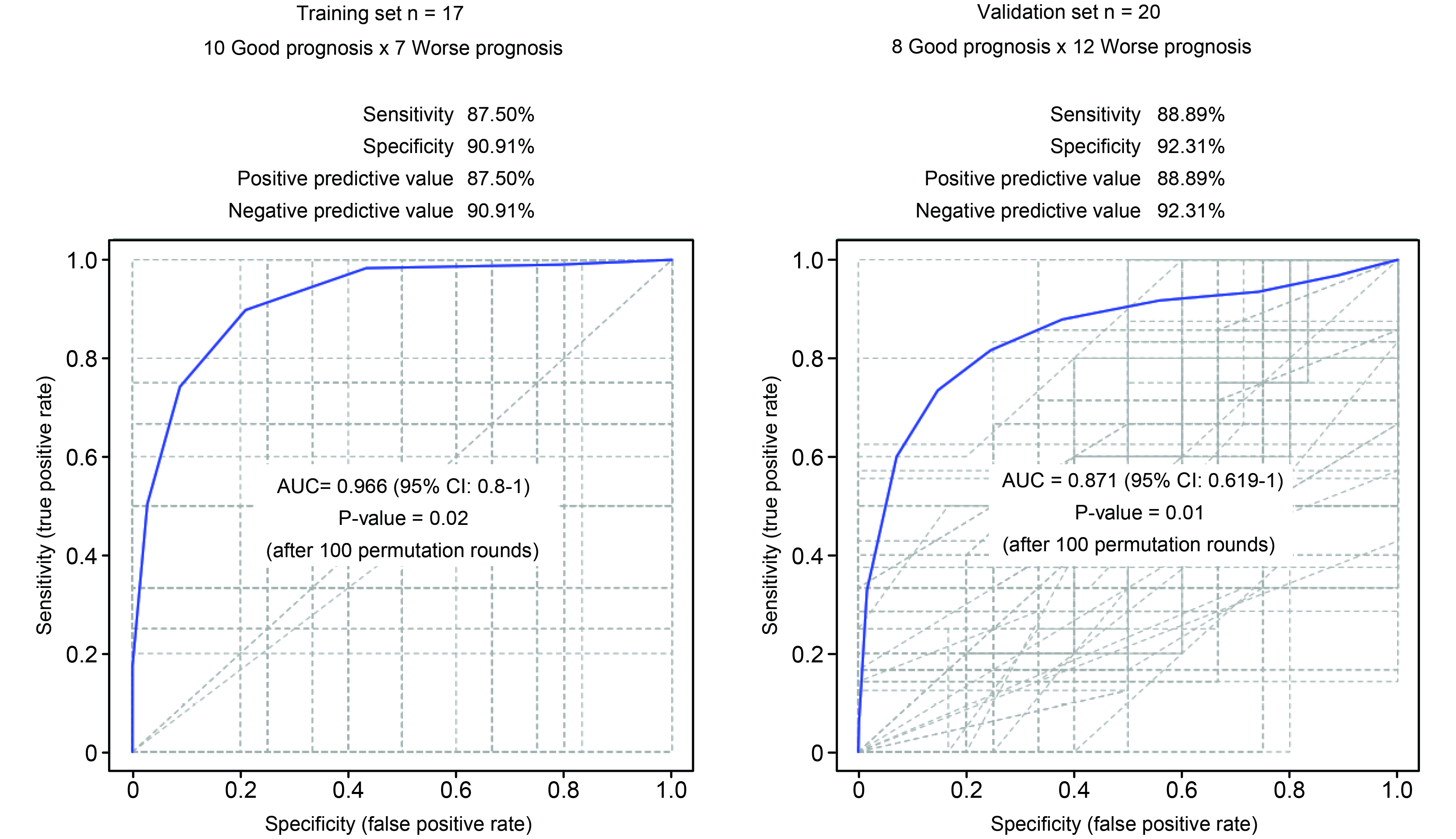

Supplement: S3 Fig — The empirical p values after 100 permutation rounds are also shown. (TIF) [file pone.0161920.s003.tif]
