# Supplementary material for: Plasma Metabolomics Biosignature According to HIV Stage of Infection, Pace of Disease Progression, Viremia Level and Immunological Response to Treatment
Source: PLoS One. 2016 Dec 12;11(12):e0161920. doi: 10.1371/journal.pone.0161920 (PMC5152829; doi:10.1371/journal.pone.0161920)
Supplement: S1 File — Supplementary tables A and B. (DOCX) [file pone.0161920.s004.docx]

**Table A. Demographics, viral loads (copies/mL of plasma) and CD4+ T cell counts. Group A= Elite controllers (EC), B= rapid progressors (RP), C= immunological responders (IR), D= INR. VL: viral loads. CMV: Citomegalovirus; HSV: Herpes Simplex Virus.**

**Table B . Summary of the measured metabolite classes and their Biological Relevance for each class.**

| **1-Amino acids**  Arginine, Glutamine, Glycine, Histidine, Methionine, Ornithine, Phenylalanine, Proline,  Serine, Threonine, Tryptophan, Tyrosine, Valine, (Iso)Leucine  *Biological Relevance*  *Amino acid metabolism, urea‐cycle, activity of gluconeogenesis and glycolysis, insulin*  *sensitivity, neurotransmitter metabolism, oxidative stress* |
| --- |
| **2-Carnitine**  C0  **3-Acylcarnitines**  C2, C3, C3:1, C4, C4:1, C5, C5:1, C6 (or C4:1‐DC), C6:1, C8, C8:1, C9, C10, C10:1, C10:2,C12, C12:1, C14, C14:1, C14:2, C16, C16:1, C16:2, C18, C18:1, C18:2  **4-Hydroxy‐ and dicarboxyacylcarnitines**  C3‐OH, C4‐OH (or C3‐DC), C5‐DC (or C6‐OH), C5‐OH (or C3‐DC‐M), C5:1‐DC, C5‐M‐DC, C7‐DC, C12‐DC, C14:1‐OH, C14:2‐OH, C16:1‐OH, C16:2‐OH, C16‐OH, C18:1‐OH  *Biological Relevance*  Energy metabolism, fatty acid transport and mitochondrial fatty acid oxidation, ketosis  oxidative stress, mitochondrial membrane damage |
| **5-Sphingomyelins**  SM C16:0, SM C16:1, SM C18:0, SM C18:1, SM C20:2, SM C22:3, SM C24:0, SM C24:1, SM C26:0, SM C26:1  **6-Hydroxysphingomyelins**  SM (OH) C14:1, SM (OH) C16:1, SM (OH) C22:1, SM (OH) C22:2, SM (OH) C24:1  *Biological Relevance*  Signaling cascades, membrane damage (e.g.neurodegeneration) |
| **7-Diacyl‐phosphatidylcholines**  PC aa C24:0/C26:0/C28:1/C30:0/C30:2/C32:0/C32:1/C32:2/C32:3/C34:1/C34:2/C34:3/C34:4/C36:0/C36:1/C36:2/C36:3/C36:4/C36:5/C36:6/C38:0/C38:1/C38:3/C38:4/C38:5/C38:6/C40:1/C40:2/C40:3/C40:4/C40:5/C40:6/C42:0/C42:1/C42:2/C42:4/C42:5/C42:6  **8-Acyl‐alkyl‐phosphatidylcholines**  PC ae C30:0/C30:1/C30:2/C32:1/C32:2/C34:0/C34:1/C34:2/C34:3/C36:0/C36:1/C36:2/C36:3/C36:4/C36:5/C38:0/C38:1/C38:2/C38:3/C38:4/C38:5/C38:6/C40:0/C40:1/C40:2/C40:3/C40:4/C40:5/C40:6/C42:0/C42:1/C42:2/C42:3/C42:4/C42:5/C44:3/C44:4/C44:5/C44:6  Biological relevance  Dyslipidemia, membrane composition and damage, fatty acid profile, activity of desaturases |
| **9-Lyso‐phosphatidylcholines**  lysoPC a  C14:0/C16:0/C16:1/C17:0/C18:0/C18:1/C18:2/C20:3/C20:4/C24:0/C26:0/C26:1/C28:0/C28:1  *Biological relevance*  Degradation of phospholipids, membrane damage, signaling cascades, fatty acid profile |
